# Comparative Genomic Analysis of *Escherichia coli* O157:H7 Isolated from Super-Shedder and Low-Shedder Cattle

**DOI:** 10.1371/journal.pone.0151673

**Published:** 2016-03-28

**Authors:** Krysty D. Munns, Rahat Zaheer, Yong Xu, Kim Stanford, Chad R. Laing, Victor P. J. Gannon, L. Brent Selinger, Tim A. McAllister

**Affiliations:** 1 Agriculture and Agri-Food Canada, Lethbridge Research Centre, Lethbridge, AB, Canada; 2 Department of Biological Sciences, University of Lethbridge, Lethbridge AB, Canada; 3 Agriculture and Forestry, Lethbridge, AB, Canada; 4 Laboratory for Foodborne Zoonoses, Public Health Agency of Canada, Lethbridge, AB, Canada; ContraFect Corporation, UNITED STATES

## Abstract

Cattle are the primary reservoir of the foodborne pathogen *Escherichia coli* O157:H7, with the concentration and frequency of *E*. *coli* O157:H7 shedding varying substantially among individual hosts. The term ‘‘super-shedder” has been applied to cattle that shed ≥10^4^ cfu *E*. *coli* O157:H7/g of feces. Super-shedders have been reported to be responsible for the majority of *E*. *coli* O157:H7 shed into the environment. The objective of this study was to determine if there are phenotypic and/or genotypic differences between *E*. *coli* O157:H7 isolates obtained from super-shedder compared to low-shedder cattle. From a total of 784 isolates, four were selected from low-shedder steers and six isolates from super-shedder steers (4.01–8.45 log cfu/g feces) for whole genome sequencing. Isolates were phage and clade typed, screened for substrate utilization, pH sensitivity, virulence gene profiles and Stx bacteriophage insertion (SBI) sites. A range of 89–2473 total single nucleotide polymorphisms (SNPs) were identified when sequenced strains were compared to *E*. *coli* O157:H7 strain Sakai. More non-synonymous SNP mutations were observed in low-shedder isolates. Pan-genomic and SNPs comparisons did not identify genetic segregation between super-shedder or low-shedder isolates. All super-shedder isolates and 3 of 4 of low-shedder isolates were typed as phage type 14a, SBI cluster 3 and SNP clade 2. Super-shedder isolates displayed increased utilization of galactitol, thymidine and 3-O-β-D-galactopyranosyl-D-arabinose when compared to low-shedder isolates, but no differences in SNPs were observed in genes encoding for proteins involved in the metabolism of these substrates. While genetic traits specific to super-shedder isolates were not identified in this study, differences in the level of gene expression or genes of unknown function may still contribute to some strains of *E*. *coli* O157:H7 reaching high densities within bovine feces.

## Introduction

*Escherichia coli* O157:H7 is a major Shiga toxin–producing foodborne pathogen that is a serious public health concern and economic problem worldwide. Healthy cattle are a primary reservoir of *E*. *coli* O157:H7, hosting the pathogen within their gastrointestinal tract (GIT) and shedding the organism into the environment via their feces. The load and frequency of *E*. *coli* O157:H7 shedding varies greatly among individual cattle [1,2]. Previous studies have reported shedding of the organism to be sporadic and of short duration [3,4] ranging from 10 to 10^7^ cfu/g feces [5]. Cattle that shed the organism at levels ≥10^4^ cfu/g of feces have been termed “super-shedders” and are reported to have a substantial impact on on-farm prevalence, transmission of and the contamination of food products with *E*. *coli* O157:H7. Matthews *et al*. [6] and Omisakin *et al*. [7] estimated that super-shedders accounted for 80 and 96%, respectively, of the total *E*. *coli* O157:H7 shed into the environment by cattle. More recently, Arthur *et al*. [8] showed that 95% of feedlot pens housing at least one super-shedder had a hide prevalence of *E*. *coli* O157:H7 of >80%, whereas only 29% of pens lacking a super-shedder exceeded this level.

Targeting super-shedders for mitigation strategies has been proposed as a means of reducing the incidence and spread of *E*. *coli* O157:H7 to pen-mates and the environment [9]. However, the specific factors responsible for super-shedding are unknown, but are presumably mediated by the characteristics of both the bacterium and the host [10]. Specifically, Arthur *et al*. [11] suggested that three components play a role in the super-shedding phenotype; i) the host genotype and phenotype, ii) the intestinal microbiome, and the iii) phenotypic and genotypic traits of the super-shedder strains of *E*. *coli* O157:H7. *E*. *coli* O157:H7 recovered from super-shedders as compared to *E*. *coli* O157:H7 from low shedders have been shown to differ in phage type [3, 12], degree of clonal relatedness [13], *tir* polymorphisms [11], presence of *stx*_2a_ and *stx*_2c_ and antiterminator Q gene alleles [11]. Although these reports have examined differences in specific genetic traits of *E*. *coli* O157:H7 strains, they have not undertaken a whole genome comparative analysis.

The objective of this study was first to determine if there are genetic differences between *E*. *coli* O157:H7 isolates obtained from super-shedder and low-shedder cattle and then compare them to other closed *E*. *coli* O157:H7 genomes reported in the literature. Phenotypic differences in carbon utilization and pH sensitivity between super-shedder and low-shedder isolates were also examined in an attempt to gain insight into specific super-shedding-associated traits that could provide insight into the development of mitigation strategies.

## Materials and Methods

### *E*. *coli* O157:H7 isolate collection, enumeration, characterization and selection

*E*. *coli* O157:H7 isolates (*n* = 10) were selected from two previous studies: Stephens *et al*. [14] whereby fecal samples were obtained from cattle (*n* = 1987) within 11 pens in two feedlots, with an average of 181 steers per pen; Munns *et al*. [3], whereby crossbred yearling feedlot steers (*n* = 400), were sampled from a single feedlot. In both studies, commercial feedlots were sampled in southern Alberta by collecting feces (50 g) through rectal palpation. All fecal samples were collected in sterile tubes, placed on ice and transported to the laboratory for analysis within 12 h. Fresh gloves were used for fecal collection from each steer within each study. Animals were handled and cared for in a manner consistent with guidelines set by the Canadian Council on Animal Care [15].

Enumeration and confirmation of *E*. *coli* O157:H7 was carried out by standard methods as described by Stephens *et al*. [14] and Munns *et al*. [3]. Super-shedders were defined as cattle that had ≥10^4^ cfu *E*. *coli* O157:H7/g feces, while low-shedders had <10^4^ cfu/g feces. A total of 658 *E*. *coli* O157:H7 isolates were collected during the first study and 126 during the second. Collected isolates were from both super- and low-shedder cattle.

All isolates (*n* = 784) were subjected to pulsed field gel electrophoresis (PFGE) as described by Munns *et al*. [3]. Isolates originating from Munns *et al*. [3] displayed only three distinct PFGE patterns. Consequently, isolates (*n* = 5) collected from super shedders in the study of Stephens *et al*. [14] were include for whole genome sequencing (Illumina, MiSeq), Biolog characterization (Biolog, California) and phage typing. This included an isolate from a steer that displayed very high super-shedding levels (K9_45; 8.45 log cfu/g feces), a distant relative/unique PFGE profile (K3_66), and isolates that displayed differential anitbiograms (data not shown). Previous research has shown that super-shedding is a transient phenomenon [3], making it difficult to identify and differentiate a super-shedder from a low-shedder. We included the low-shedding strains from Munns *et al*. [3] study due to intensive sampling (twice per day, for 8 days) that occurred within that study and thereby confirmed the low-shedding status of these individuals.

### Biolog analyses and phage typing

The phenotypic profile of isolates (*n* = 6) recovered from super-shedders were compared to those of low-shedders (*n* = 4) to determine if differences exist in carbon utilization and pH sensitivity using Omnilog phenotypic microarrays (PM) with PM1 and PM2 used for carbohydrate utilization and PM10 for pH responses (Biolog, Hayward, CA). The Biolog microarrays were conducted according to the manufacturer’s instructions. Briefly, isolates were grown on blood agar overnight at 37°C and colonies were picked with a sterile cotton swab and re-suspended in 10 mL IF-0a medium (Biolog). Cell density was then adjusted to an OD_600_ of 0.035 using a spectrophotometer (Biolog Turbidimeter) and (600 μL) of this suspension was mixed with 120 mL of IF-10a medium (Biolog), and wells in 96-well microtiter plates were inoculated with 100 μl. The plates were incubated for 48 h in the Omnilog incubator reader. At the end of the incubation period, reduction of the reporter dye was quantified using the Kinetic Plot and Parametric modules of the Omnilog Phenotype Microarray software suite and expressed as OmniLog Units. Additional statistical analysis was carried out using the "omp" package [16] for R [17] to identify differences between super-shedder and low-shedder isolates based on the area under the curve (AUC) analysis using ANOVA with significance declared at *P*<0.05.

Isolates (*n* = 10) were phage-typed (PT) at the *E*. *coli* Reference Laboratory of the Laboratory for Foodborne Zoonoses, Guelph, Ontario, using previously described procedures [18, 19] and 16 phages (numbered 1–16) that differentiated 88 PT.

### DNA sequencing, single nucleotide polymorphism (SNP) and clade typing analysis

Genomic DNA was extracted from each *E*. *coli* O157:H7 isolate (50-ng samples) and prepared for sequencing using the Blood & Cell Culture DNA Maxi Kit (Qiagen, Valencia, CA). Samples were then sequenced using MiSeq (Illumina, San Diego, CA) paired-end 100-bp sequencing. The FASTX-Toolkit was used to filter low quality reads. Paired-ends Illumina sequencing reads were then assembled into contigs using the Velvet 1.1.06 *de novo* assembler with a kmer length of 49 for all 10 strains. All genome files are available from the NCBI Genebank database (accession numbers LHAI00000000-LHA-Q00000000). Clean Illumina reads were mapped to the *E*. *coli* O157:H7 genome of reference strain Sakai [20] using Burrows-Wheeler Aligner software [21]. Sequence Alignment/Map (SAM) tools (http://samtools.sourceforge.net) were used to split, sort and merge the aligned result, and picard-tools were used to sort the binary sequence alignment data. The Genome Analysis Toolkit (GATK; [22]) was employed for base quality score recalibration, insertion and deletion (indel) realignment, duplicate removal, and SNP and INDEL discovery. In-house perl scripts were developed to select SNPs from the GATK output. Single nucleotide polymorphisms loci were annotated by using Sakai as a reference strain. For comparative purposes, we concatenated all SNPs to create a genotype for each isolate and Circos [23] was used to visualize SNP distribution among super- and low-shedder isolates and within compared genomes. A SNP tree was constructed by means of RAXML as described by Stamatakis [24] using maximum parsimony method with SNP extracted from this study combined with a previous report [25]. For each genome the SNPs were concatenated to a single alignment.

Clade typing was carried out on the basis of SNPs or combinations of SNPs specific for individual clades according to Manning *et al*. [26]. Additional comparative genomic core and accessory genomic region analyses were carried out using Panseq (http://lfz.corefacility.ca/panseq/; [27]). Stx bacteriophage insertion (SBI) site and antiterminator Q gene allele discovery was carried out as described by Besser *et al*. [4] and LeJeune *et al*. [1], respectively.

## Results and Discussion

### Isolate selection, whole genome sequencing and comparative genomics

**Fig 1:**
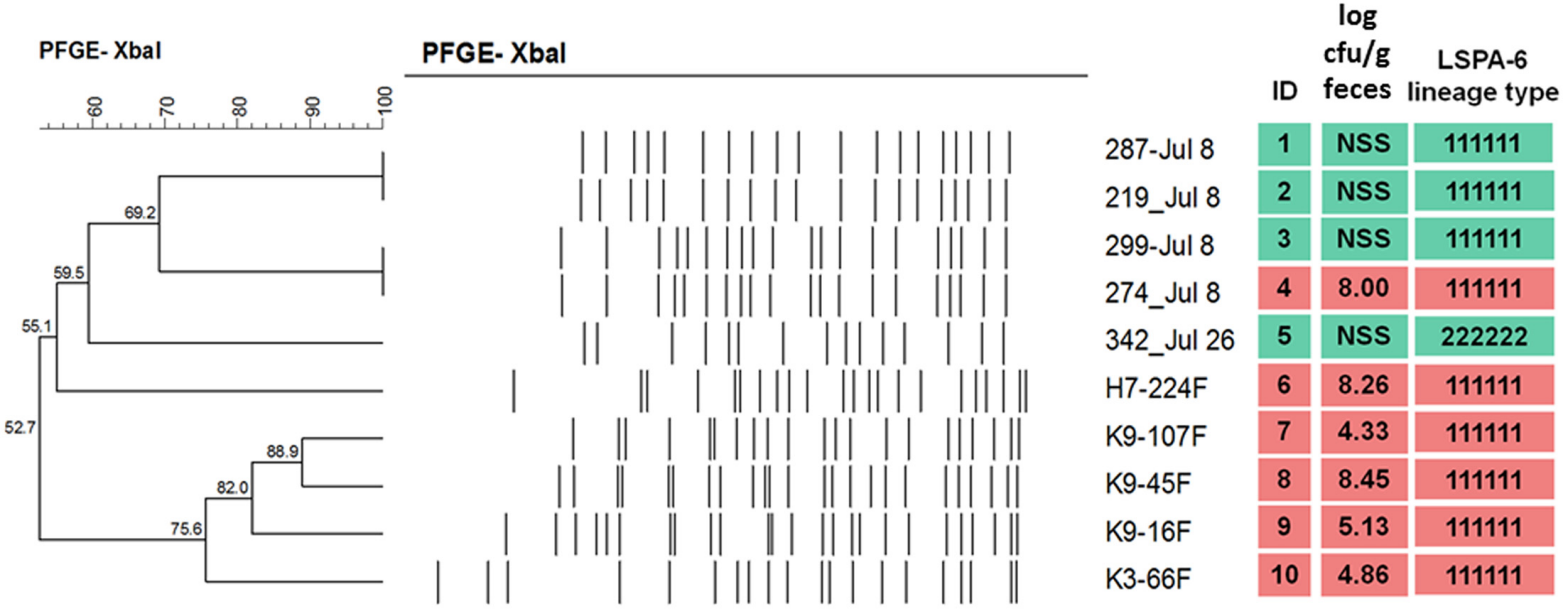
Characteristics of isolates selected for whole genome sequencing including; PFGE relatedness, shedding status (log CFU g/feces) and LSPA-6 lineage type. NSS: low-shedding.

**Fig 2:**
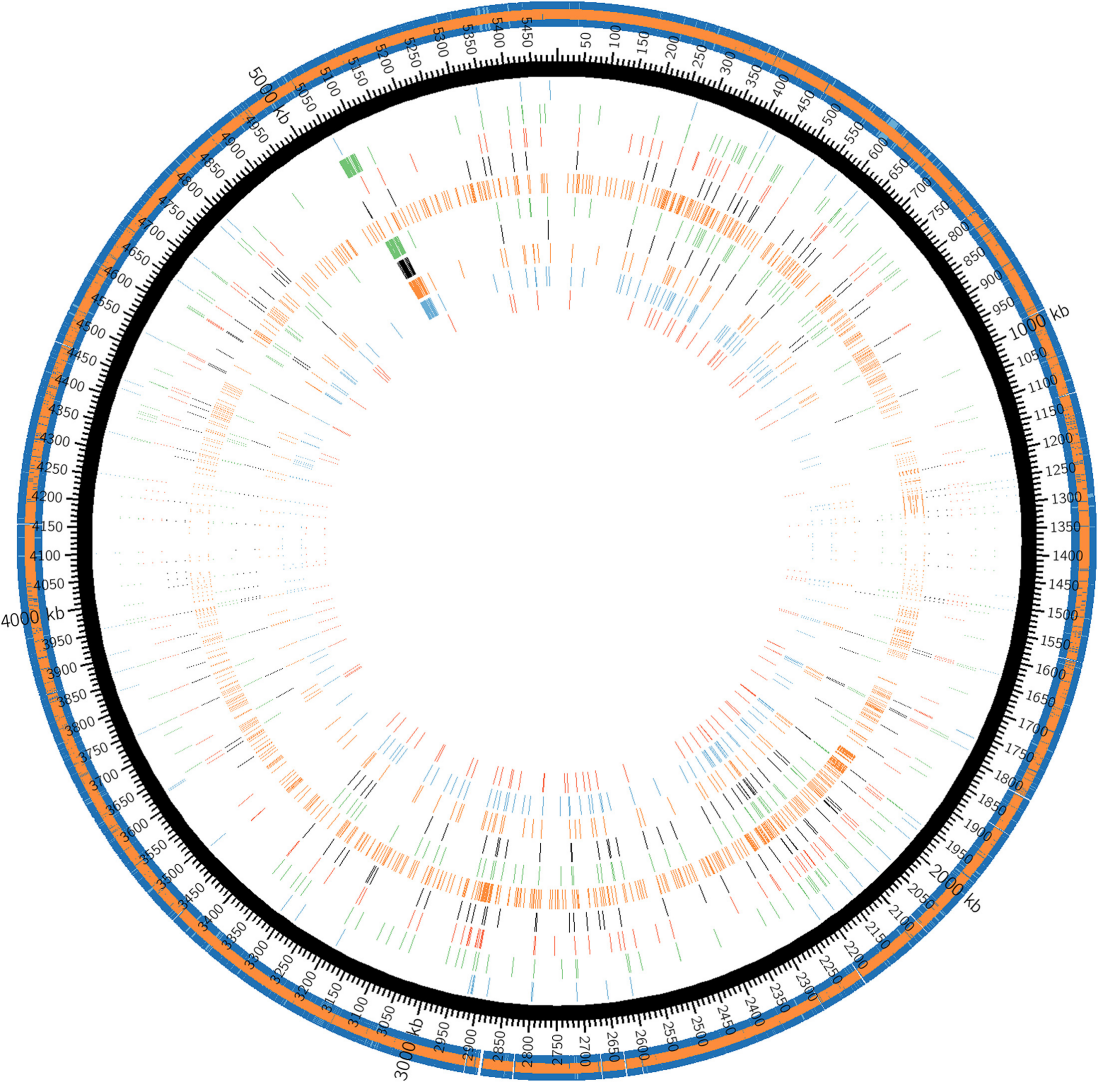
Single nucleotide polymorphisms observed within selected isolates (n = 10) as compared to references stain *E*. *coli* O157:H7 Sakai (outer ring). Isolates starting from inner circle are: K9_107F (4.33 log CFU/g), 219_Jul8 (NSS), H7_224F (8.26 log CFU/g), 274_Jul8 (8.00 log CFU/g), 299_Jul8 (NSS), 342_Jul28 (NSS), K3_66F (4.86 log CFU/g), K9_16F (5.13 log CFU/g), 287_Jul8 (NSS), and K9_45F (8.45 log CFU/g). Intensity of bands depicts frequency of SNPs.

Isolates (*n* = 6 from super-shedding cattle; *n* = 4 from low-shedding cattle) were 52.7%-100% related based on PFGE (Fig 1). One low-shedding isolate (287_Jul8) was enumerated at 2.7 log CFU/g feces at time of collection, while the other three low-shedding isolates were only recovered after enrichment and immunomagnetic separation (IMS). These isolates typed as LSPA6 lineages I (*n* = 9) and lineage II (*n* = 1) (Fig 1). Paired-end 100 bp sequencing yielded 29–30× coverage, and generated genomes composed of 231 to 368 contigs per isolate, representing 94.1 to 96.6% coverage of their genomes (LHAI00000000-LHA-Q00000000). A range of 89–2473 total SNPs were identified when sequenced isolates were compared to Sakai, the highly characterized lineage I strain of *E*. *coli* O157:H7 isolated from a 1996 outbreak in Japan (Hayashi *et al*., 2001) (NC_002695; Fig 2; Table 1). Nine of ten isolates displayed <190 non-synonymous single nucleotide polymorphisms (nsSNPs) while the only lineage II isolate (342_Jul26) exhibited >1000 nsSNP (Table 1). Super-shedder isolates, typed as linage I, from this study had far fewer (51–185) nsSNP than another super-shedder isolate (SS17; 1485 nsSNP) that was sequenced and compared to Sakai by Cote et al. [28]. This suggests that these super-shedding strains are not similar to one another and that super-shedding isolates within our study are more “Sakai-like” with SS17 resembling recently described outbreak strains (EC4115 and TW14359). Upon further examination, none of the nsSNP were consistently observed among all six super-shedding isolates. Interestingly, all of the super-shedding isolates with the exclusion of 274_Jul8 displayed an Arg→His substitution in DNA topoisomerase IV subunit A, His→Asn substitution in a cellulose synthase catalytic subunit, and a Phe→Val substitution in a host specificity protein. There were several instances where nsSNP were identified in four of the six super-shedder isolates in proteins such as lysins, exonucleases, and those involved in fructose and mannose metabolism.

**Table 1:** Number of non-synonymous SNPs, synonymous SNP and non-coding regions among super-shedding (*n* = 6), low-shedding *Escherichia coli* O157:H7 isolates (*n* = 4) and other fully closed reference genomes when compared to Sakai.

Strain	Status	Non-synonymous	Synonymous	Non-coding	Total	Reference
K9_45F	super-shedder	51	24	14	89	[14]
K9_107F	super-shedder	84	22	25	131	[14]
K3_66F	super-shedder	100	43	27	170	[14]
K9_16F	super-shedder	128	62	37	227	[14]
274_Jul8	super-shedder	138	238	17	393	[3]
299_Jul8	low-shedder	179	289	40	508	[3]
H7_224F	super-shedder	185	273	58	516	[14]
287_Jul8	low-shedder	188	301	37	526	[3]
219_Jul8	low-shedder	190	305	44	539	[3]
SS17	super-shedder	602	690	189	1481	[28]
EC4115	clinical	631	707	189	1527	[25]
TW14359	clinical	649	733	213	1595	Kulasekara et al., 2009
342_Jul26	low-shedder	1093	1070	310	2473	[29]

**Fig 3:**
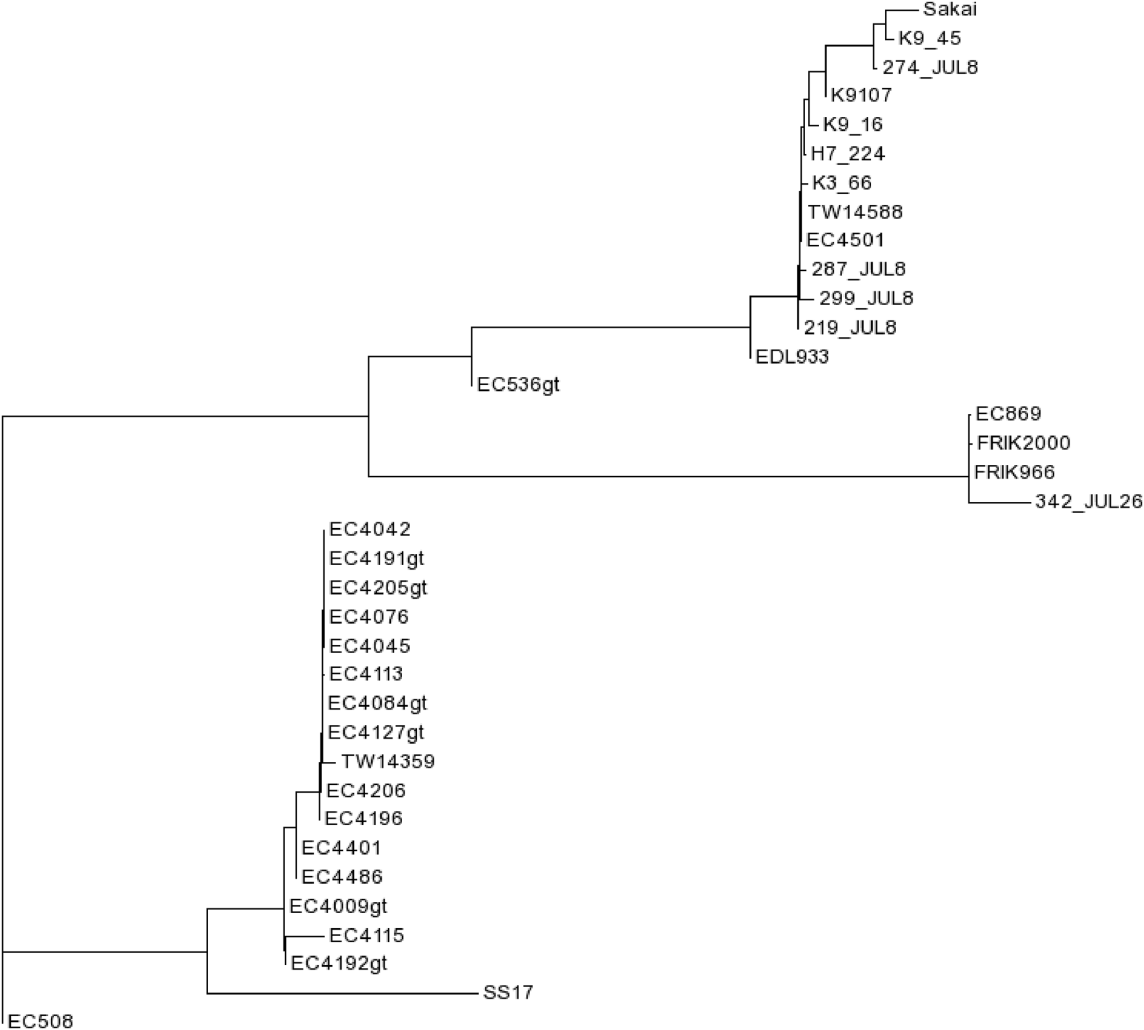
Single nucleotide polymorphism based phylogenetic tree of super-shedding (SS; *n* = 6), low-shedding isolates (NSS; *n* = 4), SS17 [28] and 25 reference *E*. *coli* O157:H7 GenBank strains.

A phylogenetic tree based on SNP differences did not provide clear genetic segregation between isolates from super-shedders *vs*. low-shedders (Fig 3). This tree also contained Genbank reference strains representing isolates obtained from human clinical cases, cattle and one complete genome isolated from super-shedding cattle from another study. Nine of the isolates were typed as lineage I after whole genome sequencing and resembled clinical outbreak strains, suggesting that isolates from both super-shedders and low-shedders can be genetically similar to isolates associated with human illness. The other isolate was a low-shedder lineage II isolate (342_Jul26) and clustered more closely with isolates of bovine rather than human origin. Arthur *et al*. [11] recently reported that isolates from super-shedders tended to share more traits in common with isolates collected from humans than isolates collected from cattle with respect to lineage and sequence variation in the *tir* allele. Using whole genome comparative analysis, Cote et al. [28] revealed a clustering of one super-shedder isolate SS17 with the lineage I/II *E*. *coli* O157:H7 isolates (TW14359 and EC4115) associated with a spinach-related outbreak.

**Fig 4:**
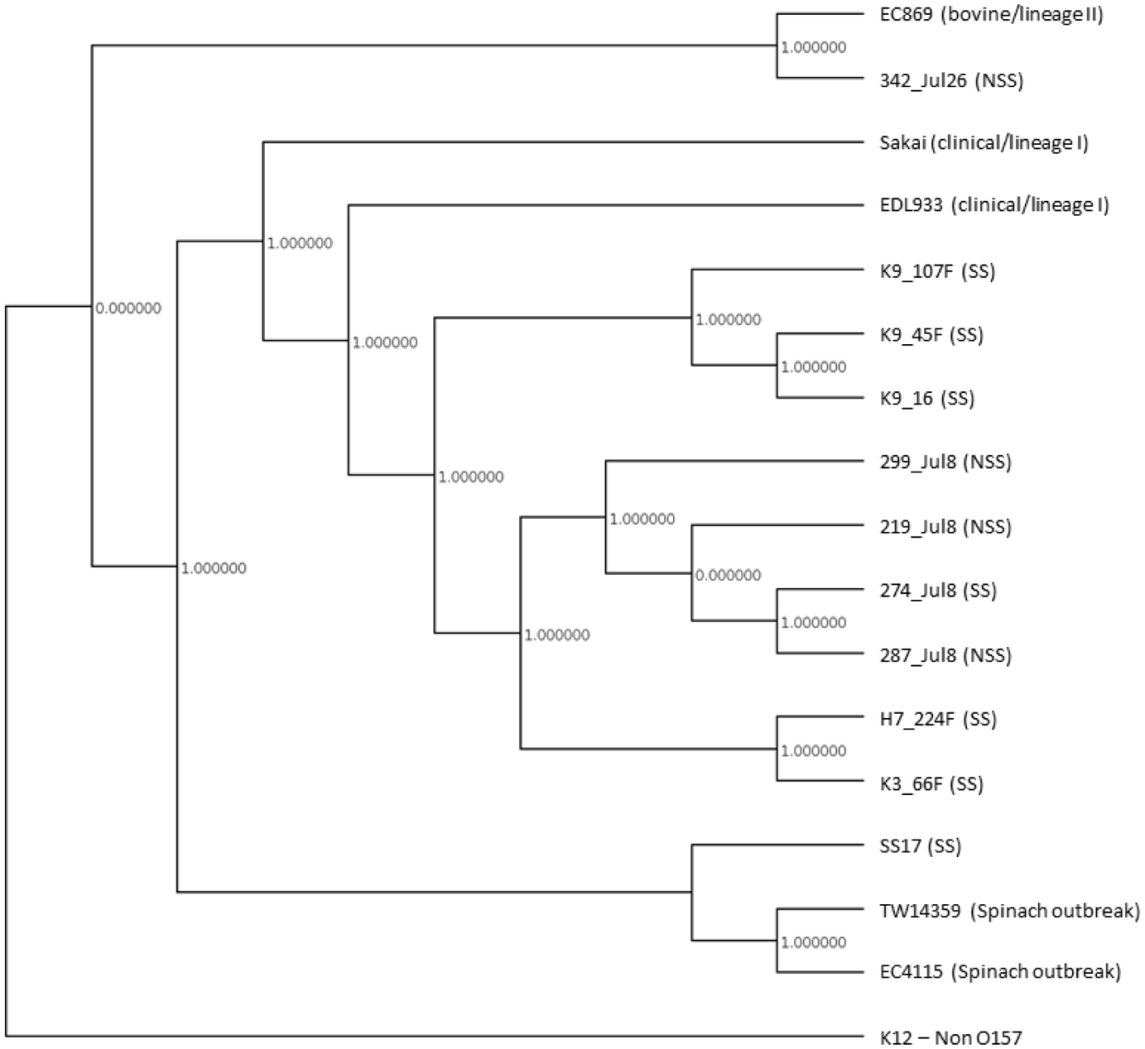
A reference free, pan-genomic comparison of super-shedding (SS; *n* = 6), low-shedding isolates (NSS; *n* = 4) and reference strains EC869 (GCA_000172035.1), Sakai (NC_002695), EDL933 (NC_002655), SS17 (Cote et al., 2015), TW14359 (NC_013008), EC4115 (NC_011353), non-O157:H7 K12 (MC1061) using Panseq.

A pan-genomic comparison of sequenced super-shedder and low-shedder isolates failed to identify any clustering based on shedding status (Fig 4), however low-shedder isolate 342_Jul 26 clustered more closely to EC869 (GCA_000172035.1), an isolate of bovine origin typed as lineage II. Further Panseq analysis did not identify differences in the absence or presence of 480 pathogenicity-related alleles between super-shedder and low-shedder isolates (data not shown). Some of these alleles have previously been used to describe the pathogenicity of strains of *E*. *coli* O157:H7 associated with human illness. Bono *et al*., [30] evaluated a non-synonymous base change (either an A or T allele) at position 255 and nucleotide repeat polymorphisms at other locations in the *tir* gene in an attempt to use these differences to delineate isolates from humans *vs*. cattle. This gene is an important virulence factor associated with the type III secretory system and plays a role in adherence of *E*. *coli* O157:H7 to intestinal epithelial cells [31]. Their study revealed that more than 99% of 108 human isolates harboured the *tir* 255 T>A T allele and lacked repeat region 1-repeat unit 3 (RR1-RU3), whereas of 77 bovine isolates, only 55% and 57% of the isolates, respectively, harboured these traits. Interestingly, Arthur *et al*., [11] reported that isolates of *E*. *coli* O157:H7 from super-shedders harbored the T allele more often (71%) than the A allele (29%). Upon examining the genomic sequences from our study, 9 of the 10 sequenced isolates (90%) harboured the T allele and lacked the RR1-RU3 region in their *tir* gene. The previously mentioned “outlier” low-shedder isolate (342_Jul26) exhibited *tir* 255 T>A A allele along with RR1-RU3. This was the same isolate that clustered more closely with isolates of bovine origin within the phylogenetic tree. A study that did not distinguish isolates based on shedding level, found isolates from cattle had equal distribution of *tir* A allele (54.8%) and *tir* T allele (45.2%), while the *tir* T allele was present in 92.9% isolates from humans [32]. The reason for the specific association of clinical isolates from humans with the T allele is unknown. The Tir protein is involved in adherence to epithelial cells and allele polymorphisms could affect cell affinity and consequently the likelihood of these isolates causing infection in humans [30]. Other important virulence genes associated with the locus of enterocyte effacement including intimin (*eae*), *espA*, *espB*, *espD* also did not show SNP differences between super-shedder and low-shedder isolates. Cote *et al*. [28] reported a number of nsSNPs in virulence and adherence genes including the adhesions *wzzB*, *fimA*, and *csgG*, along with a truncation of *cah* in a single super-shedder isolate. We did not observe nsSNPs within these genes within our isolates.

Shiga toxins are encoded by bacteriophages [33] with Stx1-, Stx2a-, and Stx2c-associated bacteriophages typically inserted within or adjacent to conserved chromosomal loci *yehV*, *wrbA* or *argW*, and *sbcB*, respectively [34,35 36]. Three principal groups of isolates sharing Stx bacteriophage insertion (SBI) site genotypes have been identified: i) isolates with a Stx2-encoding bacteriophage inserted at a location other than *wrbA* and with *yehV* occupied by a centrally truncated bacteriophage (cluster 1), ii) isolates with a Stx2-encoding bacteriophage inserted into *wrbA* and with *yehV* occupied by a truncated bacteriophage as in cluster 1 (cluster 2), and iii) isolates with a complete Stx1-encoding bacteriophage inserted into *yehV* and with a Stx2-encoding bacteriophage inserted into *wrbA* (cluster 3). Sequence data from our super-shedder and low-shedder isolates revealed that all but one of the isolates harboured the SBI cluster 3 (Table 2). One low-shedder isolate, 342_Jul26, belonged to genotype 15 and also tended to cluster with bovine isolates. This SBI genotype has also been detected in the reference strain FRIK2000 (GCA_000175755.1), a bovine isolate which does not express Shiga toxin and is part of a secondary lineage of O157:H7. Besser *et al*. [4] observed four different SBI genotypes among human isolates (*n* = 282) from the United States, with 92.5% of all isolates being represented by genotype 1 (30.1%) and 3 (62.4%). Arthur *et al*. [11] examined differences in SBI sites among *E*. *coli* O157:H7 isolates (*n* = 102) from super-shedders and found more than half (55.9%) belonged to SBI genotypes/clusters 1–3, also suggesting that SBI genotypes from super-shedders may be linked to those genotypes that are most often associated with human illness.

**Table 2:** Genetic characteristics among *Escherichia coli* O157:H7 isolates examined within this study and reference strains.

Isolate ID	Characteristic	*tir* 255 T>A polymorphism and RR1-RU3	SBI Cluster/Genotype	*Stx* presence	Anti-terminator Q gene alleles
287_Jul8	low-shedder	T–lacking RR1-RU3	Cluster 3	*stx*_*1a*_, *stx*_*2a*_	Q_933_
342_Jul26	low-shedder	A–RR1-RU3 present	15	*stx*_*1a*_, *stx*_*2c*_	Q_21_
219_Jul8	low-shedder	T–lacking RR1-RU3	Cluster 3	*stx*_*1a*_, *stx*_*2a*_	Q_933_
299_Jul8	low-shedder	T–lacking RR1-RU3	Cluster 3	*stx*_*1a*_, *stx*_*2a*_	Q_933_
K9_45F	super-shedder	T–lacking RR1-RU3	Cluster 3	*stx*_*1a*_, *stx*_*2a*_	Q_933_
H7_224F	super-shedder	T–lacking RR1-RU3	Cluster 3	*stx*_*1a*_, *stx*_*2a*_	Q_933_
274_Jul8	super-shedder	T–lacking RR1-RU3	Cluster 3	*stx*_*1a*_, *stx*_*2a*_	Q_933_
K9_16F	super-shedder	T–lacking RR1-RU3	Cluster 3	*stx*_*1a*_, *stx*_*2a*_	Q_933_
K9_107F	super-shedder	T–lacking RR1-RU3	Cluster 3	*stx*_*1a*_, *stx*_*2a*_	Q_933_
K3_66F	super-shedder	T–lacking RR1-RU3	Cluster 3	*stx*_*1a*_, *stx*_*2a*_	Q_933_
Sakai	clinical strain	T–lacking RR1-RU3	Cluster 3	*stx*_*1a*_, *stx*_*2a*_	Q_933_
EDL933	clinical strain	T–lacking RR1-RU3	Cluster 3	*stx*_*1a*_, *stx*_*2a*_	Q_933_
EC4115	clinical strain	T–lacking RR1-RU3	Cluster 1	*stx*_*2a + 2c*_	Q_21/_Q_933_
FRIK2000	bovine strain	A–RR1-RU3 present	15	*stx*_*1a*_, *stx*_*2c*_	Q_21_

The determination of SBI for a particular strain is not only dependent on where the *stx* are inserted, but also which *stx* subtypes are present [37]. Previous research has reported that strains with *stx*_2_ are more often associated with hemolytic-uremic syndrome (HUS) than strains harboring *stx*_1_ [38, 39]. These two genes have been further divided into several subtypes: *stx*_1a_, *stx*_1c_, and *stx*_1d_ for *stx*_1_, and subtypes *stx*_2a–g_ for *stx*_2_ [37]. Furthermore, the subtypes *stx*_2a_ and/or *stx*_2c_ are more often associated with HUS [40] than other *stx* types. Our data revealed that all isolates except a low-shedder isolate (342_Jul26) carried both *stx*_1a_ and *stx*_2a_, whereas 342_Jul26 carried *stx*_1a_ and *stx*_2c_ (Table 2). Arthur *et al*. [11] found that isolates from super shedders carried *stx*_2a_ or *stx*_2c_ separately more frequently than together and that *stx*_1_ was carried by half (51%) of the super-shedder strains while all isolates from low-shedders possessed the collective set of *stx*_2a_, *stx*_2c_, and *stx*_1_. Cote et al. [28] reported that their super-shedder isolate had a profile of *stx*_1_- *stx*_2_+ *stx*_2c_+ and contained two plasmids, pO157 and pSS17. Interestingly, isolates within our study carried *stx*_2a_ and *stx*_2c_, subtypes that are more often associated with increased Shiga toxin production and HUS regardless of shedding status.

The antiterminator Q gene alleles have been found to be distributed differently between human and bovine *E*. *coli* O157:H7 isolates [1] and may serve as a genetic marker for super-shedder isolates. The Q_21_/ Q_933_ PCR assay targets two different alleles of the antiterminator gene Q upstream of the prophage *stx*_2_ region [1]. Allele Q_933_ is responsible for a strong anti-terminator activity resulting in relatively high expression levels of stx_2_, while allele Q_21_ generates weak anti-terminator activity resulting in lower stx_2_ expression [41]. The Q gene may indirectly affect the colonization of bovine GIT through differences in Shiga toxin expression. *E*. *coli* O157:H7 strains harboring the Q_933_ variant of the anti-terminator gene produced significantly higher levels of stx_2_ toxin than strains with the Q_21_ variant or strains harboring both Q_933_ and Q_21_ [1, 42]. Previous research from our laboratory has shown increased levels of stx_2_ enhances adherence of *E*. *coli* O157:H7 to the intestinal epithelium in cattle [43]. In our study, all but one isolate harboured the Q_933_ allele with one low-shedder isolate (342_Jul26) containing the Q_21_ allele (Table 2). Interestingly, 59.8% of super-shedding isolates examined by Arthur *et al*., [11] harboured the Q_933_ either alone (41.2%), in combination with Q_21_ (18.6%), suggesting that Q_933_ may be play a role in enabling *E*. *coli* O157:H7 to be shed at super-shedder level through increased stx_2_ expression. It is important to note that the similarity among super-shedding and low-shedding strains within this study may be a reflection of the fact that isolates were chosen from only two studies, albeit from different years (2007 and 2011) and different feedlots in southern Alberta.

### Carbon utilization profile and phage type (PT) of *E*. *coli* O157:H7 from super-shedders and low-shedders

*E*. *coli* O157:H7 possess multiple metabolic pathways to oxidize a range of carbon sources, making it difficult to equate differences in metabolism to a single metabolic pathway. If differences in carbohydrate metabolism among strains of *E*. *coli* O157:H7 could be linked to increased fitness, it may provide insight into the factors that promote the survival and proliferation of super-shedder strains within the GIT. Franz *et al*. [44] found higher oxidization rates of propionic acid, α-ketobutyric acid, and α-hydroxybutyric acid among *E*. *coli* O157:H7 strains that survived for a 211 *vs*. 70 days in manure-amended soil. In our study, no significant differences in the oxidation of these substrates were observed between super-shedders and low shedder isolates. However, galactitol, 3-O-β-D-galactopyranosyl-D-arabinose and thymidine, displayed increased oxidation in super-shedder as compared to low-shedder isolates (Table 3).

**Table 3:** Differences in average *Escherichia coli* O157:H7 oxidative activity towards various carbon sources between super-shedder (*n* = 6) and low-shedder (*n* = 4) isolates as measured by Biolog phenotypic microarrays. Bold values represent higher oxidation rates.

		Area under the curve analysis using kinetic data (Omnilog units)		
Carbon Source	Key genes involved in metabolic pathways	Super-shedder isolates (mean±SEM; n = 6)	Low-shedder isolates (mean±SEM; *n* = 4)	*P*-value
Galactitol	*gatY*,*gatZ*, *gatA*, *gatB*, *gatC*, *gatD*, *gatR*	**4416** ± 141	1292 ± 24	0.00068
Thymidine	*deoA*, *deoB*, deoC, deoD, deoR, yegT	**5688** ± 134	3415 ± 78	0.032
3-O-β-D-Galactopyranosyl-D-Arabinose	*hns*, *ebgA*, *ebgB*, *ebgC*, *ebgR*	**4305** ± 168	2178 ± 79	0.014
D-Raffinose	*rafY2*, *lacY*	2593 ± 98	**5649** ± 179	0.0012
L-Methionine	*metN*, *metI*, *metQ*, *metK*	229 ± 8	**657** ± 11	0.051
N-Acetyl-D-Galactosamine	*aga/gam regulon (agaR*, *kbaZ*, *agaV*, *agaW*, *agaE*, *agaF*, *agaA*, *agaS*, *kbaY*, *agaB*, *agaC*, *agaD*, *agaf)*	2801 ± 134	**5150** ± 201	0.013

Galactitol/tagatose transport involves the *gatYZABC* operon consisting of *gatY*, *gatZ*, *gatA*, *gatB*, and *gatC* as well as two genes, *gatD* and *gatR*, located immediately downstream of *gatYZABC* operon. This operon encodes for tagatose bisphosphosphate aldolase, tagatose-6-phosphate kinase, phosphotransferase system (PTS) as well as galactitol-specific transporter subunits IIA, IIB and IIC. The *gatD* gene encodes for galactitol-1-phosphate dehydrogenase and the *gatR* is the repressor of the galactitol utilization operon. Upon examining our sequence results, no sequence differences were observed between super-shedders and three of four low-shedder isolates. The outlier, low-shedder strain (342_Jul26) exhibited an amino acid substitution Ile95Val in the *gatC* gene. This substitution may not contribute to a difference in galactitol utilization as both isoleucine and valine are non-polar amino acids, raising the possibility that neither the secondary or tertiary structure were altered.

A cytoplasmic protein, dH-NS, plays a role in the expression of many genes either directly or indirectly by modifying the condensation of chromosomal DNA. Previous research has demonstrated that when the *hns* structural gene and upstream regulatory regions were replaced with a mutant cassette, O-β-D-galactopyranosyl-D-arabinose was no longer metabolized. Genetic differences within *hns* between super-shedder and low-shedder isolates within the study were not observed. The *ebg* gene cluster of *E*. *coli* O157:H7 consists of *ebgA*, *ebgB*, and *ebgC*, which encodes for the subunits of the ebg ß-galactosidase (evolved- ß-galactosidase; EbgA), and *ebgR* which encodes for the operon repressor. The natural substrate of this operon is unknown [45], however its involvement in catabolizing galactopyranosyl-D-arabinose has been reported [46]. Three of the four low-shedder isolates and one super-shedder isolate exhibited an amino acid substitution Arg871His in EbgA. The remaining super-shedder isolates and one low-shedder isolate (342_Jul26) harbored arginine at this position, a substitution also observed in two strains (EDL933, Sakai) isolated from outbreaks in humans. Arginine and histidine are both positively charged and whether this mutation has an impact on the utilization of O-β-D-galactopyranosyl-D-arabinose requires further investigation.

Thymidine catabolism in *E*. *coli* involves a number of enzymes including thymidine phosphorylase (*deoA*), 1,5 phosphodeoxyribomutase (*deoB*), deoxyribose-5-phosphate aldolase (*deoC*) and nucleoside phosphorylase (*doeD*). The expression of the operon associated with these genes is regulated by the *deoR* repressor and a nucleoside transporter protein (*yegT*) that may play a role in thymidine uptake in *E*. *coli*. The sequence of these genes and their promoter regions were identical between super-shedder and low-shedder isolates, and thus differences in thymidine metabolism between super-shedder and low-shedder isolates could not be attributed to differences in the sequence of these genes.

In contrast, N-acetyl-D-galactosamine (Aga), D-raffinose and L-methionine displayed significantly higher rates of oxidation in low-shedder than super-shedder isolates (Table 3). The phosphoenolpyruvate:carbohydrate PTS is an enzyme complex responsible for the transport of a large number of different types of carbohydrates in bacteria [47]. A gene cluster encoding the Aga PTS and other catabolic enzymes are responsible for transport and catabolism of Aga. *E*. *coli* O157:H7 strains typically display an Aga+ phenotype, however, isolates recovered from a 2006 spinach-associated *E*. *coli* O157:H7 outbreak displayed an Aga^−^ phenotype [48]. Sequence alignment of the 11,745-bp *aga/gam* cluster of genes in EDL933 with those in the Sakai strain and isolates from the 2006 spinach outbreak revealed a single nucleotide difference (G:C→A:T) in the *agaF* coding for EIIA^Aga/Gam^, changing a conserved glycine residue to serine (Gly91Ser). Upon analyzing *agaF* in our sequenced isolates we found no differences between super-shedder and low-shedder isolates.

Raffinose is a trisaccharide composed of galactose, glucose, and fructose and was oxidized in all the isolates in this study, but to a lesser extent in super-shedder isolates (Table 3). The plasmid born Raf operon in generic *E*. *coli* has been well characterized [49] and contains genes coding for proteins involved in the uptake and utilization of raffinose including an α-galactosidase (*rafA*), raffinose permease (*rafB*), and sucrose hydrolase (*rafD*) as well as the repressor (*rafR*). Downstream of the *rafRABD* operon, *rafY* encodes a glycoporin involved in the uptake of maltose, sucrose, and raffinose [50]. The product of *rafY* impacts the transport of raffinose through LacY. No homologs of the *rafRABD* gene cluster are found in *E*. *coli* O157:H7, however a *rafY* homolog annotated as *rafY2* were present in the genomes of both super-shedder and low-shedder isolates and displayed no sequence differences.

Methionine is one of two sulfur-containing amino acids and plays a role in a variety of methyltransferase reactions as a precursor of S-adenosyl-methionine (SAM). S-adenosylmethionine is synthesized from methionine and ATP by SAM synthetase (*metK*). As with raffinose, L-methionine was oxidized to a lesser extent in super-shedder than low-shedder isolates (Table 3). The methionine transport system includes *metN*, *metI* and *metQ* encoding for ATPase, permease and substrate binding protein, respectively. No sequence differences were identified in genes related to methionine metabolism among super-shedder and low-shedder isolates.

**Fig 5:**
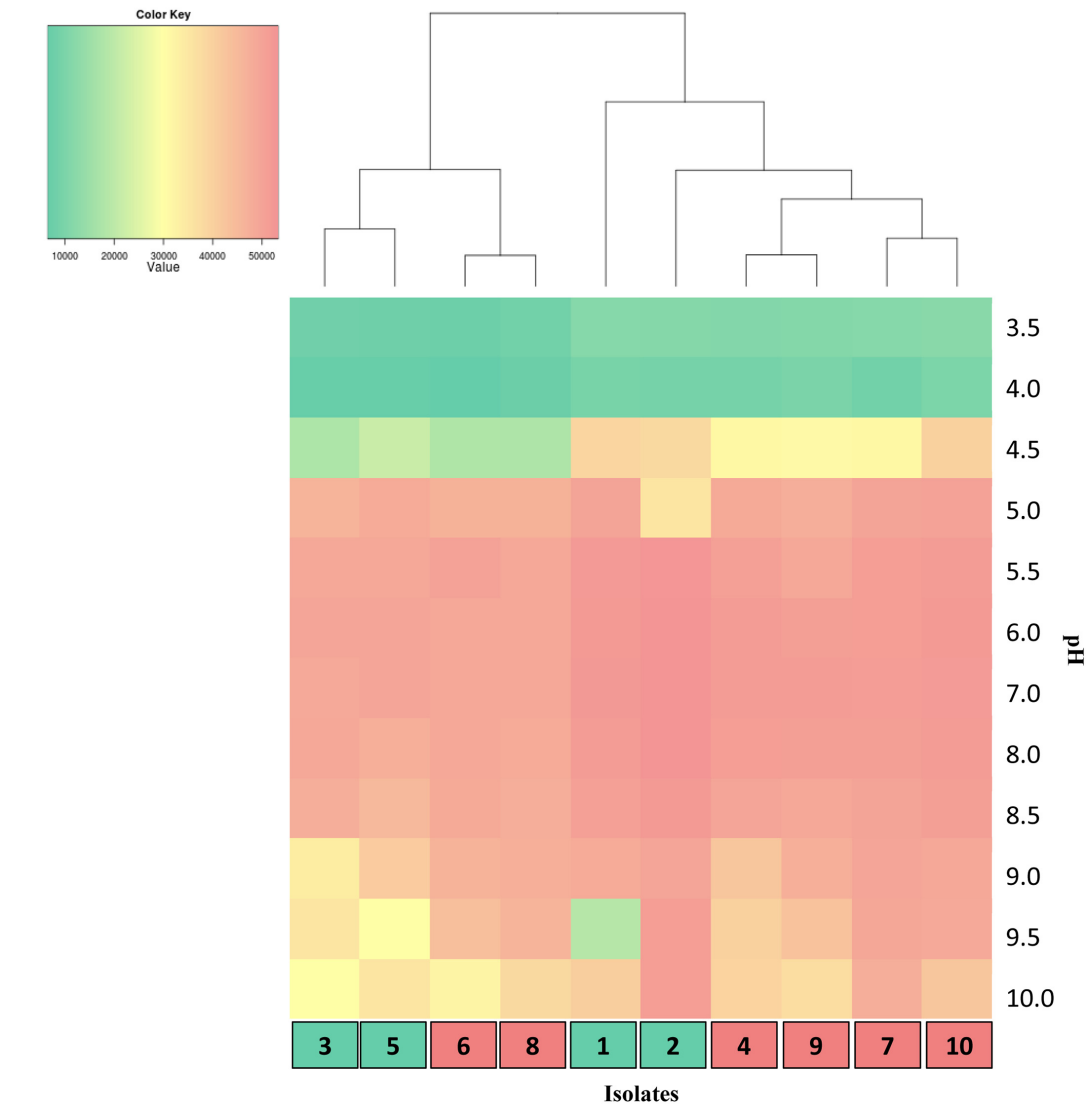
Growth of isolates over a pH range from 3.5–10. Isolates were grown for 48 h at various pH ranging from 3.5 to 10. The colour key represents relative growth based on oxidative activity using area under the curve (Omnilog units). Isolates are identified in pink (super-shedders) and teal (low-shedders) boxes as in Fig 1.

A critical element in the emergence of *E*. *coli* O157:H7 was the evolution of acid resistance under positive selective pressure within the GIT of ruminants, an attribute that promotes survival in acidic environments [51] and may result in increased infection and proliferation within the GIT of cattle fed high grain diets [52]. When super-shedder and low-shedder isolates were grown over a pH range from 3.5 to 10, super-shedder isolates had a significant (P = 0.01) greater increase in oxidation activity at pH 4.5 (Fig 5) and a slight increase at 9.5 (P = 0.08) as compared to low-shedders. The UPGMA dendrogram obtained after 48 h showed two distinct branches; however, this was not related to shedding status or from which study isolates were collected (Fig 5).

If super-shedder isolates have a greater ability to survive in a more acidic GIT environment, then perhaps these strains are more likely to proliferate and achieve super-shedder levels, especially in cattle fed grain-based diets. *E*. *coli* O157:H7 achieves acid resistance using three systems, of which the glutamate decarboxylase (gad) system is the most efficient and has been shown to be essential for the organism’s ability to colonize the intestinal epithelium in cattle and to protect against oxidative stress [53]. No sequence differences were observed among genes known to be involved in the glutamate decarboxylase acid-resistance system including *gadA*, *gadB*, *gadBC*, and *gadX*. Additional genes that play a role in the maintenance of cell wall integrity (*tolQ* and *tolR*), oxidative stress resistance (*soxS)*, osmoregulation (*proP* and *proB*), as well as several genes encoding proteins involved in DNA repair and protein turnover (*uvrA*, *uvrB*, *uvrC*, *uvrY*, *ruvB* and *mfd*) and molecular chaperones (*msrB/yeaA*, *grpE*, *ybbN*, *ybiY*, *hslO*, *clpS* and *clpA*) were examined and no SNP differences were identified between super-shedder and low-shedder isolates.

It is important to note that genes that exhibit identical sequences may still be differentially expressed. *E*. *coli* O157:H7 genomes contain several open reading frames (ORFs) encoding for proteins of unknown function. Further investigation of ORFs of unknown function could elucidate their role, if any, in the differential utilization of compounds examined within this study along with their possible role in the super-shedder phenotype. In addition, several unidentified/hypothetical genes associated with regions corresponding to O-islands and S-loops [20, 54] in *E*. *coli* O157:H7 genomes may be involved in the uptake and utilization of the substrates that displayed differential oxidization in this study. Therefore, along with genetic sequencing, comparative expression microarray or RNAseq-based gene expression assays from isolates grown in identical conditions may aid in deciphering differences in overall gene expression profiles between super-shedder and low-shedder isolates. However, it would be challenging to perform these comparative studies in real-time with samples collected from the GIT considering the apparent transient nature of super-shedding [3]. Simulating the environmental conditions within the GIT to differentiate expression levels between super-shedder and low-shedder isolates also presents challenges.

All the isolates recovered from super-shedders were PT14a, whereas those from low-shedders included phage type PT91 (219 Jul_8), PT43 (342_Jul26) and PT14a (*n* = 2). Previous research has identified five different PTs (PT8, PT14a, PT21, PT33, and PT34) among the *E*. *coli* O157:H7 isolates collected within Canada with PT14a and PT8 being most common (Jokinen *et al*., 2011). In contrast, Arthur *et al*. [11] examined super-shedder isolates (*n* = 102) from the United States and identified 19 different phage types with PT4 accounting for 30% of isolates. Another study found an association between PT21/28 and super-shedders within a population of cattle in Scotland [12, 55]. Interestingly, PT4 is the most common phage type associated with *E*.*coli* O157:H7 clinical outbreaks strains in the United States, where as PT21/28 and PT14a are the most common phage types among clinical strains isolated from Scotland [56] and Canada, respectively [57]. This suggests that super-shedders may harbour those strains that are most frequently associated with human illness.

### Clade typing

Manning *et al*. [26] genotyped more than 500 clinical strains of *E*. *coli* O157:H7 based on 96 SNPs that separated strains into genetically distinct clades and identified a ‘hyper-virulent’ clade (clade 8) of *E*. *coli* O157:H7 among isolates obtained from a 2006 outbreak associated with raw spinach in the United States. Clade 8 strains have been shown to have 2-fold greater adherence to bovine epithelial cells (MAC-T) and increased expression of virulence genes, including those that are LEE- (*espAB*, *tir*, *eae*, *stx*_*2*_) and plasmid encoded (*hlyA*, *toxB*, *tagA*) [58]. One study isolated an *E*. *coli* O157:H7 strain from a super-shedder which was genotyped as clade 8 [28]. Due to the fact that only a single isolate was examined this finding may not be specific to the super-shedder phenotype for *E*. *coli* O157:H7. Our study is the first to examine six super-shedding isolates all of which were clade 2. This suggests that super-shedding isolates from our study are not directly related to this hyper-virulent strain.

### Biofilm formation

In a previous review [10] it was hypothesized that super-shedder isolates may have a superior ability to form biofilms on the intestinal epithelium. Biofilm sloughing in the intestine could be responsible for the fecal densities of *E*. *coli* O157:H7 that are required for the host to be designated a super-shedder. Intermittent sloughing of the intestinal biofilm could also account for the sporadic nature of super-shedding in cattle. Previous research, using DNA microarrays, discovered that 79 genes, representing 1.84% of the *E*. *coli* genome, were differentially expressed during biofilm formation as compared to planktonic growth [59]. Among these genes, three involved in adhesion and auto aggregation, several encoding structural proteins such as OmpC, OmpF and OmpT, and *slp* (encoding an outer-membrane lipoprotein induced after carbon starvation) showed increased expression in biofilms. Some of these genes (*slp* and *ompC*) have been associated with the initial steps of *E*. *coli* biofilm formation on abiotic surfaces [60, 61] However, no differences in the sequence of these genes were observed between super-shedder and low-shedder isolates in our study. To date, most bacterial adherence assays have been standardized using cell lines derived from human cancers such as HeLa, HEp-2 and Caco-2 [62, 63]. These cell lines may not accurately reflect the mechanistic processes involved in the adherence of bacteria to cattle GIT epithelial cells. Kudva and Nystom [64] standardized a protocol for a RAJ squamous epithelial (RSE) cell–bacterial adherence assay. One study reported *in vitro* analysis of adherence profiles of one super-shedder isolates’ ability to bind and adhere to this cell line [28]. Results showed that all of the RSE cells exposed to the super-shedder isolate had a significantly higher number of bacteria/cells as compared to RSE cells exposed to a control strain, EDL933 [28]. This suggests that super-shedder isolates may have a superior capacity to adhere to bovine rectal epithelial cells.

### 342_Jul26 Anomaly

As described throughout the manuscript, one low-shedder isolate (342_Jul 26) consistently clustered separately from the other nine isolates displaying genetic characteristics that were more closely related to bovine than clinical or outbreak lineages (Table 2). This ‘bovine-like’ isolate (342_Jul26), typed as clade 5, SBI genotype 6, displayed differences in carbon utilization, exhibited *tir* 255 T>A A allele with RR1-RU3 repeating unit, and carried *stx*_1a_ and *stx*_2c_ as well as the Q_21_ variant of the anti-terminator Q gene alleles. This isolate also displayed a much higher number of nsSNP (n = 1093) than the other nine isolates when compared to the outbreak strain Sakai (Table 1). Whereas the remaining three low-shedders as well as all super-shedder isolates were more ‘clinical-like’; typing as clade 2, SBI cluster 3, lacking the *tir* polymorphism and RR1-RU3, possessing *stx*_1a_ and *stx*_2a_ and the Q_933_ variant.

To further investigate this observation, we characterized additional isolates (*n* = 78; 3 isolates each from 26 isolations) from Munns et al. [3]. These isolates were co-cultured at the same time as all of the sequenced isolates described in this study. Twenty-one isolates were recovered from steers that were excreting super-shedding levels of *E*.*coli* O157:H7. Twenty-seven were isolated after enrichment and IMS detection. The remaining isolates (*n* = 30) were recovered from steers that were shedding an enumerable amount of *E*. *coli* O157:H7 but not at super-shedding levels. All isolates were screened for *stx* genes anti-terminator Q variants and *tir* allele genotype using PCR and sequencing as described by Besser et al. [4]. Although, not as comprehensive as whole genome sequencing, results showed all additional isolates screened as lineage I, displayed ‘clinical-like’ characteristics lacking *stx*_*2c*_ and displaying Q_933_ insertion site for the antiterminator alleles (data not shown).

Seventy five of those isolates, with the exception of 2 isolates that were co-isolated from 342_Jul26 sample, possessed the *tir* 255 T>A T and lacked the RR1-RR3 regions. Interestingly, the remaining isolates collected from animal 342 at all other sampling dates (*n* = 18), (before and after Jul26), were “clinical-like”. It appears that during the sampling of 342_Jul26, when the steer was at a low-shedding level, the ‘bovine-like’ population of *E*. *coli* O157:H7 was in a high enough abundance to be preferentially isolated. This observation emphasises that sampling over a long period of time could reveal dynamics of multiple populations present within a single host. In addition, it can be reasoned that although the ‘clinical-like’ strains of *E*. *coli* O157:H7 may be present in a host at any given time in low numbers, host factors including the microbial community within the GIT as well as the composition of the diet may play a role in their colonization and likelihood of achieving sufficient density for super-shedding to occur.

## Conclusions

This study undertook a whole genome comparative analysis of *E*. *coli* O157:H7 isolates collected from super-shedders and low-shedders in an effort to identify specific genes and or changes in gene sequence that may be linked to the super-shedding state. Although these were not identified, it is important to note that there may be multiple factors, in addition to the pathogen itself, which could contribute to super-shedding. Previous studies have provided evidence that the microbiomes of a super-shedder host differ from low-shedding hosts [65, 66]. Future studies that aim to further understand acid resistance of super-shedding isolates, differential expression of genes associated with biofilm formation or microbiomes of the bovine host may enable effective mitigation strategies to be identified.
